# Sensitivity analysis for time-to-event data accounting for intra-individual variability in time-varying covariates with missing data

**DOI:** 10.1038/s41598-025-09599-3

**Published:** 2025-07-24

**Authors:** Madiha Liaqat, Luciana Chiapella, Pradeep Mishra, Walid Emam, Yusra Tashkandy, Adelajda Matuka

**Affiliations:** 1 University of the Punjab, Lahore, Pakistan; 2 Institute of Pharmacology, Faculty of Medicine, University of Buenos Aires – CONICET, Buenos Aires, Argentina; 3 College of Agriculture, JNKVV, Rewa, India; 4 Department of Statistics and Operations Research, College of Science, King Saud University, P.O. Box 2455, Riyadh, 11451 Saudi Arabia; 5 Department of Economics, Faculty of Economics, University of Bologna, Bologna, Italy

**Keywords:** Event time data, Multiple imputation, Sensitivity analysis, Delta adjustment, Ecology, Evolution, Anatomy, Diseases, Risk factors

## Abstract

The Delta-Adjusted (DA) approach in multiple imputation (MI) is applied under several key assumptions in the Cox hazard model, where two time-dependent covariates have missing observations. Missingness in these covariates is assumed to be not missing at random (NMAR) and is modeled through delta adjustments, with different delta values specified to capture deviations from the missing at random (MAR) assumption. Within the MI framework, missing values are imputed under various plausible missingness scenarios while preserving the relationship between time-dependent covariates and the event-time outcome. Event-time dependence is accounted for by assuming that missingness in covariates is influenced by an individual’s treatment response or disease progression, thereby capturing intra-individual variability. Compared to other sensitivity analysis techniques, DA under MI explicitly adjusts imputed values using delta shifts, providing a structured approach to handling NMAR data. Unlike traditional methods that rely on pattern-mixture or selection models without direct imputation, DA generates multiple datasets with controlled sensitivity adjustments, ensuring a better variability assessment. Additionally, DA allows flexible assumptions regarding loss to follow-up (FU) and event occurrence through delta values, whereas other methods often rely on fixed assumptions about missingness. Its results are more interpretable, providing sensitivity bounds for treatment effects under different missing data scenarios.

**Supplementary Information:**

The online version contains supplementary material available at 10.1038/s41598-025-09599-3.

## Introduction

Longitudinal studies, which collect data from individuals over time, often encounter the challenge of loss to FU, leading to incomplete data. Missing data due to FU loss can compromise internal validity if significant differences exist between participants who complete the study and those lost to FU.

Several factors contribute to missing data, including missed visits, unrecorded measurements, or administrative errors. In medical research, patients are typically observed until the study period ends, but some may drop out prematurely, especially when dealing with time-dependent variables. Cox regression models are widely used for analyzing event-time data while accommodating censoring from FU loss or the end of the observation period. These models require complete covariate data; however, missing time-dependent covariates introduce substantial challenges for accurate modeling and inference.

Researchers implement well-designed studies, train personnel, and adopt alternative data collection strategies to mitigate missing data^1^. Despite these measures, missing data remains prevalent, particularly in longitudinal studies where dropout rates increase over time. If not appropriately addressed, missing data can lead to biased and inefficient estimates^2^.

The impact of missing data on event-time analysis depends on the missing data mechanism. Little and Rubin^3^ classified missing data into three categories: MCAR, where the missing data occur by chance and are unrelated to observed and unobserved data; MAR, which is related to the observed data but not the unobserved data; and MNAR, which depends on unobserved data.

MCAR and MAR are often considered ignorable, whereas MNAR is non-ignorable and introduces a higher risk of bias^4,5^. More than 5% of missing data requires appropriate analytical methods to ensure valid estimates^6^. Ignoring systematic differences between participants who drop out and those who complete the study can result in severely biased outcomes^7^. To mitigate these issues, researchers often employ a combination of missing data handling techniques and conduct sensitivity analyses to evaluate the impact of MNAR data^3,8^.

Loss to FU in longitudinal studies creates two distinct participant populations, which may differ systematically, introducing bias in complete case analysis (CCA)^9^. Under the ignorable MAR assumption, imputation techniques can provide asymptotically unbiased estimates. However, distinguishing between MAR and MNAR is challenging based solely on observed data^10^. Failing to account for this distinction threatens internal validity, making it essential to apply appropriate missing data methods and conduct sensitivity analyses to ensure robust study outcomes^11^.

MI is a widely used method for handling missing covariates in regression models. Typically, MI incorporates additional covariates in the imputation model to estimate missing values while maintaining alignment with the outcome model across imputed datasets. However, a significant challenge arises when the imputation model and the substantive model are incompatible, potentially leading to biased results.

To address the issue, Van Buuren^12^ proposed incorporating event-related variables: event indicator, the event or censoring time, and the logarithm of time into the imputation model. This approach, initially applied to fixed-in-time covariates within a Cox proportional hazards model, improved imputation accuracy by ensuring consistency with the outcome model. Later adaptations by Clark and Altman^13^ and Barzi and Woodward^14^ further refined this technique by varying predictor selection based on study requirements.

Burne and Abrahamowicz^15^ introduced an alternative imputation method using martingale residuals, incorporating outcome information directly. This approach leverages the martingale residual, which quantifies an individual’s excess event risk at a given time, improving imputation reliability.

For time-to-event analyses with multiple incomplete covariates, various MI techniques have been developed. These include multivariate normal imputation via Markov Chain Monte Carlo (MCMC) and iterative univariate covariate regression imputation using Multiple Imputation by Chained Equations (MICE), also known as Fully Conditional Specification (FCS).

Building upon these methodologies, this study introduces the DA- MI approach to address missing data in time-dependent covariates within Cox regression models. The DA-MI approach explicitly adjusts imputed values using sensitivity parameters, providing a structured method to handle NMAR data. By integrating sensitivity analysis into the imputation framework, DA-MI systematically accounts for deviations from the MAR assumption, ensuring more accurate estimates in the presence of non-ignorable missingness.

This study applies DA-MI within the MICE framework to examine the relationship between time-dependent covariates and time-to-tumor shrinkage in prostate cancer (PC) using Cox regression modeling. By addressing missing data issues, this analysis aims to enhance clinical decision-making and improve patient care outcomes.

The objective of this paper is to propose a more systematic approach to incorporating a time-to-event outcome within an imputation model, specifically addressing scenarios where the outcome is assumed to follow a proportional hazards model. The presented methods focus on the case of a single incomplete variable; however, similar challenges arise when dealing with multiple incomplete variables. The paper is structured as follows: Sect. 2 describes the materials and methods. Section 3 presents the results of the PC dataset analysis. Section 4 focuses on sensitivity analysis, and Sect. 5 concludes with a discussion of the findings and their implications.

## Methods

A common approach to handling missing data is CCA, where cases with missing values are excluded. While CCA is unbiased under the MCAR assumption, it reduces statistical power by discarding data. Under the MAR assumption, CCA produces biased estimates, and it is highly inappropriate for NMAR cases, where missingness depends on unobserved values^16^.

Single imputation replaces missing values with a single estimate, such as the mean, median, or predicted value. Although this method retains all individuals in the analysis, it can produce biased results when missing data involves multiple variables or different sources of missingness. Additionally, single imputation does not fully account for the uncertainty surrounding the imputed values, leading to invalid estimates, particularly under MAR and NMAR conditions^17^.

MI overcomes the limitations of CCA and single imputation by creating multiple datasets, replacing missing values with randomly sampled values from a predictive distribution based on observed data. Each dataset is analyzed separately, and Rubin’s rules combine estimates to account for uncertainty in missing data. Particularly effective under MAR, MI systematically addresses differences between observed and missing values, preventing the power loss associated with CCA^18^. The choice of a missing data method depends on the missing data mechanism. MI is commonly applied when missingness follows MAR, where the probability of missing data depends only on observed variables. By drawing values from predictive distributions, MI enhances statistical inference validity, retaining all individuals in the analysis and reducing parameter estimate bias^18^.

However, when missingness follows an MNAR mechanism, where the probability of missing data depends directly on unobserved values, MI alone is insufficient. In such cases, DA is necessary to account for deviations from the MAR assumption. DA modifies imputed values by introducing an offset parameter (δ), which adjusts the imputed missing data to reflect plausible departures from MAR^19,20^. This approach allows for sensitivity analyses, providing a systematic framework to assess how varying degrees of departure from MAR influence results.

The choice between MI and MI with DA depends on the pattern of missingness in the data. MI alone is appropriate when missingness is driven by observed covariates and can be reliably estimated using available data. MI with DA is necessary when missingness is related to unobserved factors, requiring sensitivity analyses to ensure robustness. This study employs MI to address MAR-based missingness and extends it to MI with DA to evaluate NMAR scenarios, ensuring a comprehensive assessment of the impact of missing data in prostate cancer analysis.

### Model-based approaches

MI is an alternative approach to handling missing data that accounts for uncertainty by imputing missing observations with plausible values under the MAR assumption. The imputation model relies on observed data to estimate multiple plausible values, creating multiple complete datasets. These datasets are analyzed individually, and the results are combined to obtain valid statistical inferences.

Selection Models (SeM) and Pattern Mixture Models (PMMs) are applied using Bayes’ theorem to assess the effect of different magnitudes of the NMAR mechanism. Since the NMAR mechanism depends on assumptions and distributions beyond the observed data, appropriate modeling is necessary.

SeM developed by Heckman^21^ assumes a mechanism that predicts completeness and multiplies response weights by the marginal outcome distribution. Rubin^18^ and Little^3^ introduced PMMs, which model missingness as a function of observed data. A Bayesian prior is required to address the unknown component, selected based on external data, expert knowledge, or literature.

Zhao et al.^22^developed a sensitivity analysis method for handling missing outcomes in event-time data under the MAR assumption and deviations from MAR. That approach uses the Kaplan-Meier (KM) estimator and Cox PH model^23^incorporating MI to estimate potential event times for patients who withdrew from the study.

This study employs MI under DAto compare the effects of MCAR, MAR, and NMAR mechanisms.MCAR is analyzed using CCA, while MAR is handled using MI. Departures from MAR are addressed by introducing an offset parameter to adjust imputed$\:{Y}^{mis}$ based on the observed data distribution $\:{Y}^{obs}$. Multiple versions of the MAR-imputed dataset are used to explore different NMAR scenarios.

### Sensitivity analysis

Sensitivity analyses are crucial in follow-up studies to evaluate the robustness of estimates based on missing data assumptions. These analyses should be pre-specified, and all study participants must be included. Starting with the MAR assumption, sensitivity analyses assess deviations from MAR^24^. Primary analyses should be conducted under the MAR assumption, while sensitivity analyses should explore NMAR scenarios. Given the presence of missing data, distinguishing between MAR and NMAR using available data alone is challenging. If results under MAR and NMAR do not differ substantially, the analysis is considered robust.

This study applies CCA assuming MCAR, where the loss to FU is unrelated to the outcome or confounders. Individuals completing the FU period are assumed to be a random sample from the original study population. Under the MAR assumption, individuals lost to FU are considered exchangeable with those who completed FU, given observed covariates. MI is used to impute missing values in the prostate cancer dataset.

Under NMAR, loss to FU depends on the outcome variable, making individuals lost to FU non-exchangeable with those who remained in the study. To address this, the MAR imputation model is modified by incorporating DA, providing a flexible approach for imputing univariate data under NMAR conditions^19^.

The MAR-based imputation model is:

$$\:{Y}_{ij}={\beta\:}_{0}+{\beta\:}_{1}{X}_{ij}+{u}_{oj}+{\epsilon\:}_{ij},$$

1 $$\:{u}_{oj}={\psi\:}_{00}+{\psi\:}_{01}{W}_{j}+{v}_{oj}$$

Where, $\:{Y}_{ij}$ is the observed outcome for individual i at time j, $\:{\beta\:}_{0}$is intercept term, $\:{\beta\:}_{1}$ is coefficient for predictor variable $\:{X}_{ij}$​, which represents effect of covariate on the outcome Y. $\:{X}_{ij}$is predictor for individual I at time j. $\:{u}_{oj}$is random effect specific to group j, capturing the unobserved heterogeneity. The residual error term $\:{\epsilon\:}_{ij}$is associated with individual i at time j, assumed to be independently distributed. For the random effect model, $\:{u}_{oj}$the random effect associated with group j. $\:{\psi\:}_{00}$is the fixed intercept termfor random effect model.$\:{\psi\:}_{01}$ is coefficient for covariate$\:{W}_{j}$ in random effect model, which affects $\:{u}_{oj}$. $\:{W}_{j}$ is a covariate specific to group j, influencing the random effect.$\:{v}_{oj}$ is residual random effect for group j, assumed to be independently distributed.

And imputation model under NMAR is given as,

2 $$\:{Y}_{ij}={\beta\:}_{0}+{\beta\:}_{1}{X}_{ij}+\delta\:\left(1-{r}_{ij}\right)+{u}_{oj}+{\epsilon\:}_{ij},\:$$.

where$\:{Y}_{ij}$ is observed outcome for individual i at time j,$\:{\beta\:}_{0}$ is intercept term, $\:{\beta\:}_{1}$ is coefficient for$\:{X}_{ij}$. $\:\delta\:$is parameter representing effect of non-random missingness (NMAR). It adjusts the model for the departure from MAR by accounting for the missing data mechanism.$\:r=1$ for observed $\:Y$, and $\:r=0$ for missingness.$\:{u}_{oj}$is random effect for group j, and $\:{\epsilon\:}_{ij}$ is residual error term^20^.This MI under the DA approach can be further refined by allowing δ to vary across individuals with missing data.

## Applied data example

This study focuses on PC patients, with observations post-therapy for Alkaline Phosphatase and PSA, both of which are recognized as significant biomarkers for disease progression^25^. Baseline variables include continuous factors such as Age and BMI, and categorical variables such as Gleason Score and Drug usage. The sample consists of 1,504 patients diagnosed with primary PC at Mayo Hospital, Lahore, followed from registration until tumor shrinkage or loss to FU. Exclusion criteria include patients lacking post-therapy monitoring for Alkaline Phosphatase and PSA, those with incomplete PSA data post-therapy, and those missing baseline variables.

Among the 1,504 patients enrolled between 2012 and 2019, tumor shrinkage occurred in 18.5% of patients with complete data and 64.5% of those with missing data. In this study, the event indicator represents tumor shrinkage, modeled using a Cox proportional hazards (PH) model to assess the association between various covariates and time to tumor shrinkage^23^.Tumor shrinkage is a critical clinical endpoint in prostate cancer studies, often used to assess treatment response and disease progression. It is typically evaluated using imaging techniques and biomarker measurements, with a reduction in tumor size indicating a positive response to therapy^26^. The model includes baseline covariates such as Age (in years), Body Mass Index (BMI), Gleason Score, Drug, and Tumor Grade, along with two time-dependent covariates, prostate-specific antigen (PSA) and Alkaline Phosphatase.Treatment options include External Beam Radiation Therapy (EBRT), Androgen Deprivation Therapy (ADT), prostatectomy, and combinations of these therapies.By incorporating time-dependent effects, the model captures dynamic changes in biomarker levels and their influence on the hazard of tumor shrinkage, providing a comprehensive understanding of PC progression and treatment response.

3 $$\begin{aligned}\lambda\:\left(\frac{t}{X}\right) & ={\lambda\:}_{o}\left(t\right)\text{e}\text{x}\text{p}({\beta\:}_{1}Age+{\beta\:}_{2}GleasonScore+{\beta\:}_{3}BMI+{\beta\:}_{4}PSA+{\beta\:}_{5}Drug \\ & \quad + {\beta\:}_{6}Alkaline\:Phasphatase\left({t}^{(obs,mis}\right))\end{aligned}$$.

where,$\:{\lambda\:}_{o}\left(t\right)$ is the baseline hazard function, which represents the hazard when all covariates are 0. X= {Age, Gleason Score, BMI, PSA, Drug, Alkaline Phosphatase(t)} represents the covariates.

Since Alkaline Phosphatase has missing values, the model needs to account for this. MI is employed for the missing values of Alkaline Phosphatase and treated as a time-dependent covariate within the Cox model.

For the missing alkaline phosphatase, the model is written as:

4 $$\begin{aligned}\lambda\:\left(\frac{t}{X,{A}_{AlkalinePhosphatase}}\right) & ={\lambda\:}_{o}\left(t\right)\text{exp}\left({\beta\:}_{1}Age+{\beta\:}_{2}GleasonScore+{\beta\:}_{3}BMI+{\beta\:}_{4}PSA \right. \\ & \quad \left. +{\beta\:}_{5}Drug+{\beta\:}_{6}AlkalinePhosphatase\left(t\right) \right)\end{aligned}$$.

Where,$\:{A}_{\:AlkalinePhosphatase}$ represents the imputed values of Alkaline Phosphatase over time.

Analysis began with MI under the MAR assumption to fill in missing observations. Missing measurements in Alkaline Phosphatase are imputed using a linear model based on Platelets, Age, BMI, Drug (therapy), PSA and Gleason Score, with missing data grouped by a missingness indicator variable. MI is conducted using chained equations (MICE), with 5 datasets and 5,000 iterations^27^.

For non-MAR (NMAR) scenarios, the MAR-imputed values are adjusted by multiplying them by a constant.Alternatively, adding a constant to the MAR-imputed values is another approach for NMAR.Analysis of Alkaline Phosphatase is applied to NMAR-imputed datasets to estimate predictors and calculate 95% confidence intervals (CIs). Model fitting is performed on each imputed dataset, and Rubin’s rules^18^are used to combine the results.

**Fig. 1:**
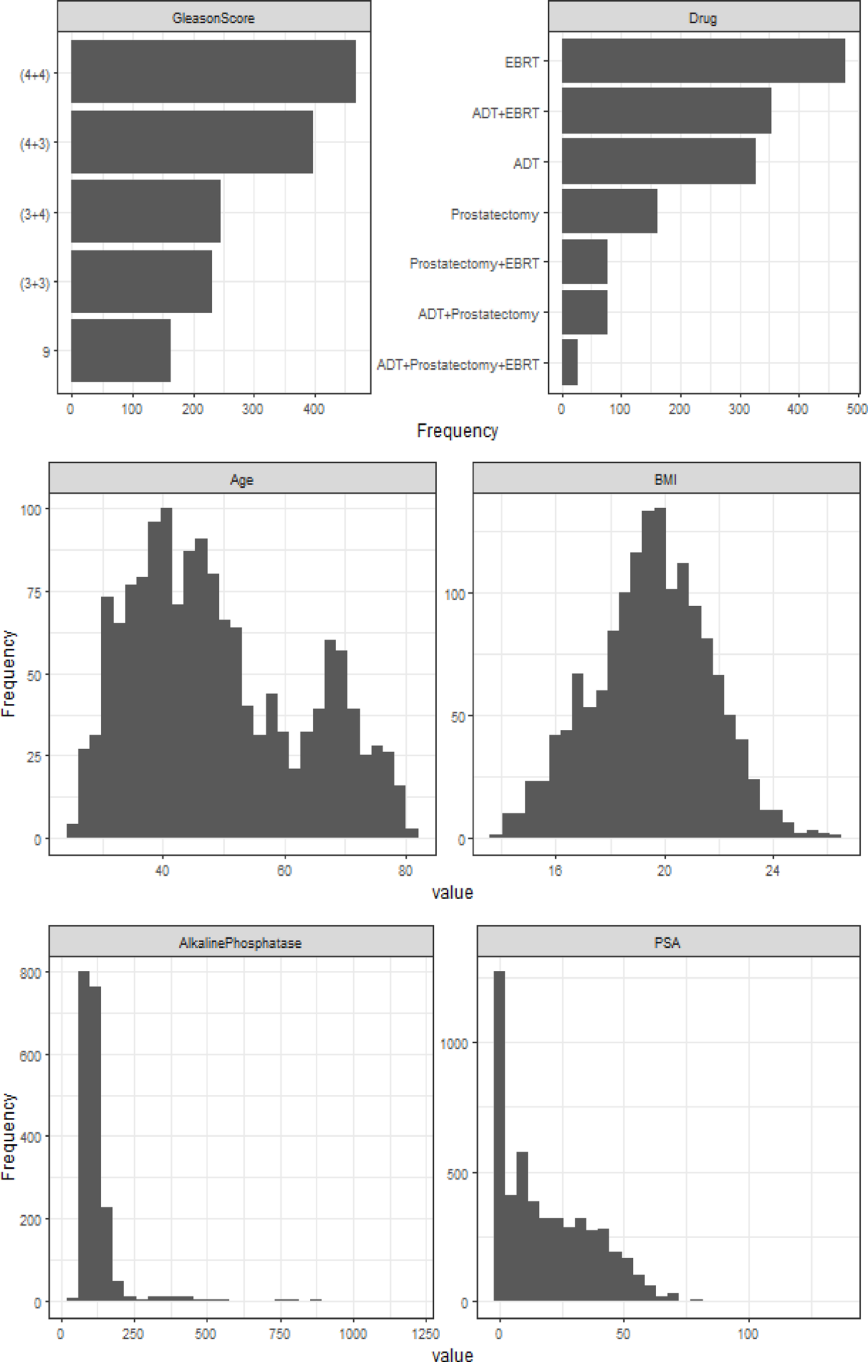
Distribution of variables in prostate tumor dataset.

Figure 1 presents an exploratory analysis of the variables in the PC dataset. The quantitative variables Alkaline Phosphataseand PSA exhibit markedly asymmetric distributions, while BMI follows a symmetric distribution, and Age shows a bimodal pattern. Among the 1,504 patients, 960 (63.8%) experienced prostate tumor shrinkage during the study follow-up period. The median time to tumor shrinkage is 8 months, with a mean ± standard deviation of 7.93 ± 2.93 months. ForAlkaline Phosphatase, only 27 patients had complete follow-up data, and 61.6% (3,083/5,008) of the observations are missing.

**Fig. 2:**
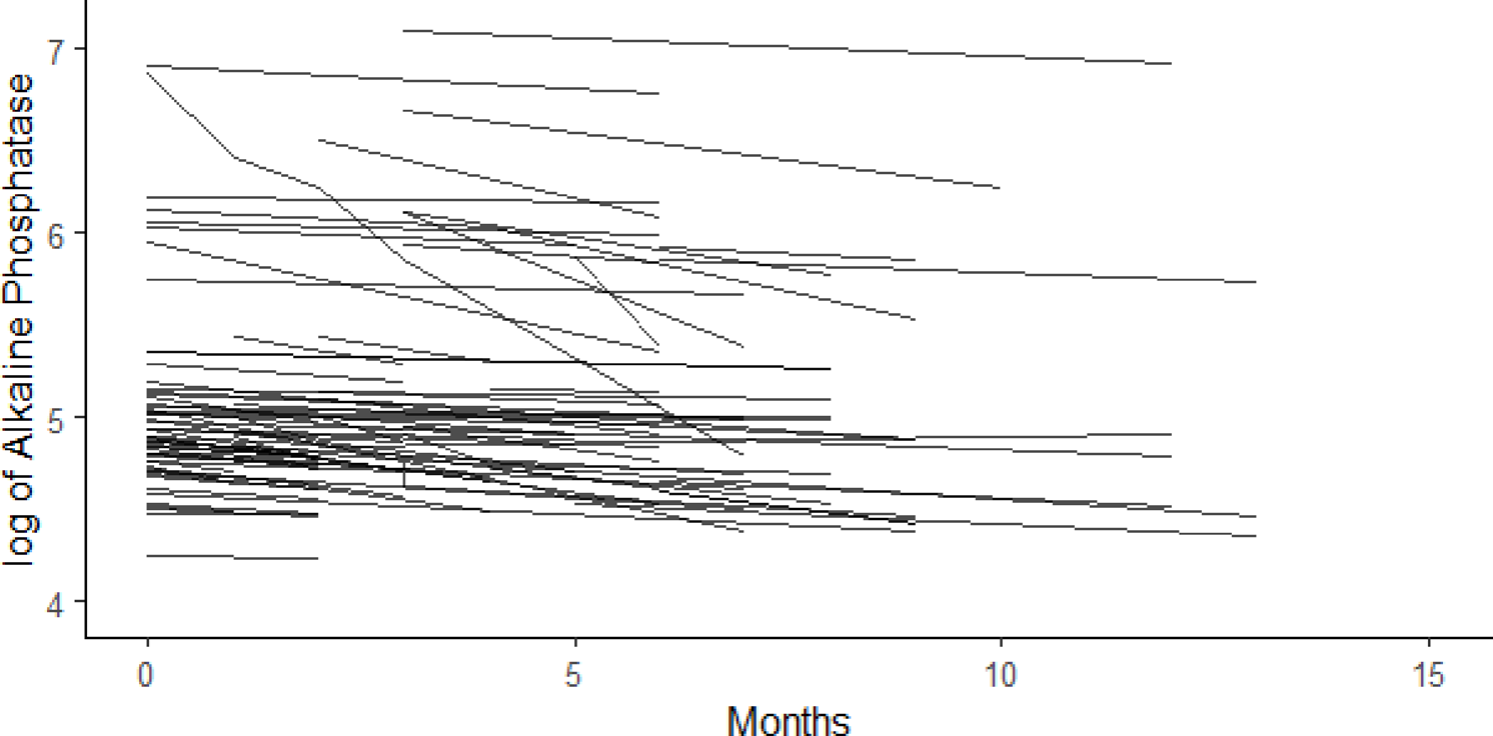
Longitudinal behavior profiles of the logarithm of Alkaline Phosphatase, by patient.

Figure 2 illustrates the longitudinal behavior of the logarithm of Alkaline Phosphatasebased on observed data. It shows a linear trend over time, with random slopes and intercepts for each individual, capturing individual variability. This suggests that, on average, Alkaline Phosphataselevels change consistently over time in PC patients, though some patients exhibit steeper changes in Alkaline Phosphataselevels than others.

**Fig. 3:**
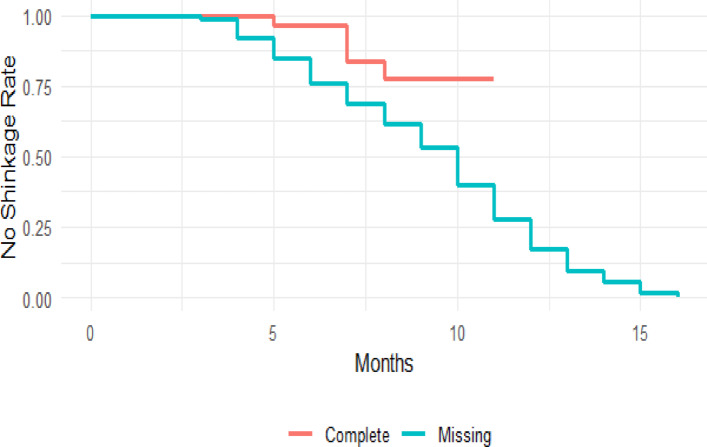
Kaplan-Meier curves for patients with complete Alkaline Phosphatase values ​​in all follow-ups (“Complete” group) and patients with at least one missing value (“Missing” group).

Figure 3 illustrates Kaplan-Meier (KM) survival curves, comparing tumor shrinkage probabilities between patients with complete and missing Alkaline Phosphatasedata. The KM curve for patients with completeAlkaline Phosphatase data suggests a more gradual decline in tumor presence, whereas those with missing Alkaline Phosphatasedata exhibit an earlier onset of tumor shrinkage. This discrepancy indicates potential selection bias, where missingness may be associated with more aggressive disease progression or inconsistent FU.

To address these biases, MIisapplied using chained equations (MICE), incorporating key covariates such as Age, BMI, Gleason Score, PSA, Drug, and the Nelson-Aalen estimator of cumulative baseline hazard^28^. The imputed values are analyzed using Cox regression, yielding hazard ratios (HRs) that are comparable to, but slightly attenuated compared to, those from the complete-case analysis. This supports the robustness of MI in mitigating bias while underscoring the importance of sensitivity analyses.

Once the imputations are performed, the logarithm of Alkaline Phosphatasevalues is reconverted to its original scale, and a Cox regression model is fitted using Age, BMI, Gleason Score, and Drug as fixed covariates, with PSA and Alkaline Phosphataseas time-dependent covariates. Each imputed dataset is analyzed individually, and results are combined using Rubin’s rules.

**Fig. 4:**
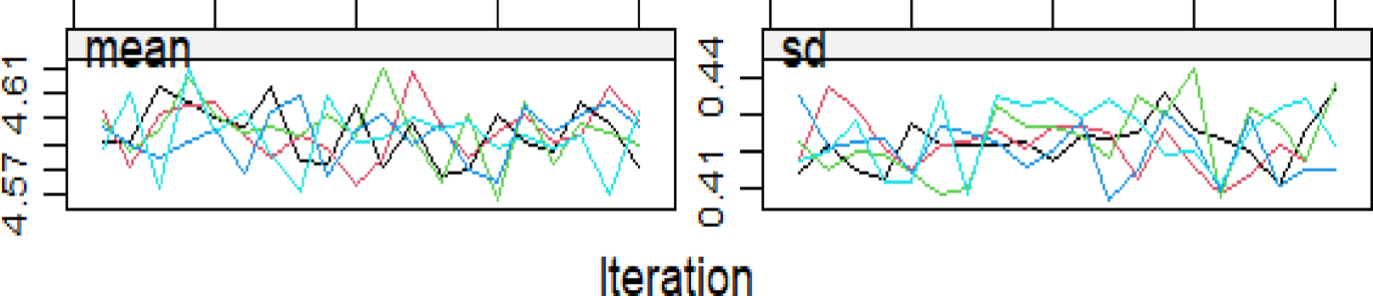
Iteration history for each imputation run for the logarithm of Alkaline Phosphatase.

Figure 4 presents the iteration history for each imputation run related to logarithm-transformed Alkaline Phosphatasevalues. The iterative process demonstrates the convergence of the imputation algorithm, which is crucial for ensuring the accuracy and efficiency of the MI process. The stability of parameter estimates across iterations indicates a consistent solution and reliable imputed values.

Table 1 presents a comparison of patient characteristics based on whether Alkaline Phosphataselevels are recorded or missing. Significant differences are observed across multiple variables, highlighting potential systematic differences between the two groups. Patients with recorded Alkaline Phosphatasevalues are significantly older (mean ± SD age: 65.41 ± 11.54 years) than those with missing Alkaline Phosphatasevalues (48.65 ± 13.72 years, *p* < 0.001). Similarly, the BMI was lower in the complete data group (18.45 ± 2.44) compared to the missing data group (19.54 ± 2.13, *p* = 0.009). The distribution of Gleason Scores differed significantly (*p* < 0.001) between the two groups. Patients with available Alkaline Phosphatasedata have a higher proportion of aggressive disease, as 48.1% had a Gleason Score of 9, compared to only 10.2% in the missing data group. Conversely, lower Gleason Scores (e.g., 3 + 3 and 3 + 4) are more prevalent in the missing data group.

**Table 1:** Descriptive measures of the recorded variables, depending on complete or missing data for the alkaline phosphatasevariable.

Variable	AlkalinePhosphatase		*p*-value
	Complete (*n* = 27)	Missing (*n* = 1477)	
Age (mean (SD))	65.41 (11.54)	48.65 (13.72)	< 0.001
Gleason Score (n, (%))			
(3 + 3)	3 (11.1)	228 (15.4)	< 0.001
(3 + 4)	3 (11.1)	242 (16.4)	
(4 + 3)	2 (7.4)	395 (26.7)	
(4 + 4)	6 (22.2)	461 (31.2)	
9	13 (48.1)	151 (10.2)	
BMI (mean (SD))	18.45 (2.44)	19.54 (2.13)	0.009
PSA (median (Q1;Q3))	25.10(10.40;39.40)	9.12 (0.92;26.30)	< 0.001
Drug (n, (%))			< 0.001
ADT	0 ( 0.0)	328 (22.2)	
ADT + EBRT	4 (14.8)	349 (23.6)	
ADT + prostatectomy	9 (33.3)	69 ( 4.7)	
ADT + prostatectomy + EBRT	0 ( 0.0)	27 ( 1.8)	
EBRT	6 (22.2)	472 (32.0)	
Prostatectomy	8 (29.6)	153 (10.4)	
Prostatectomy + EBRT	0 ( 0.0)	79 ( 5.3)	
Status			< 0.001
Low	5 (18.5)	952 (64.5)	
High	22 (81.5)	525 (35.5)	

PSA levels are markedly higher in patients with complete Alkaline Phosphatasedata (median: 25.10, Q1: 10.40, Q3: 39.40) than in those with missing Alkaline Phosphatasevalues (median: 9.12, Q1: 0.92, Q3: 26.30, *p* < 0.001). This suggests that higher PSA levels is associated withAlkaline Phosphatase observability. Treatment distributions also showed significant differences (*p* < 0.001). Among patients with recordedAlkaline Phosphatase, ADT + Prostatectomy (33.3%) and EBRT (22.2%) are the most common treatments. In contrast, patients with missing Alkaline Phosphatasevalues are more frequently treated with EBRT alone (32.0%) or ADT (22.2%). Notably, some treatment combinations (e.g., Prostatectomy + EBRT) are absent in the complete data group.

The proportion of patients with high-risk tumor status is significantly higher among those with recorded Alkaline Phosphatase(81.5%) compared to those with missing Alkaline Phosphatasevalues (35.5%, *p* < 0.001). Conversely, the low-risk category is dominant in the missing data group (64.5%) but much lower in the complete data group (18.5%).

The results indicate that missingness in Alkaline Phosphataseis not random and is associated with key clinical variables such as age, Gleason Score, PSA levels, treatment type, and tumor status. Patients with complete Alkaline Phosphatasedata tend to be older, have higher Gleason Scores, elevated PSA levels, and more aggressive disease, which reflect a selection bias whereAlkaline Phosphatase is more frequently recorded in patients with advanced prostate cancer. This pattern highlights the importance of addressing missing data mechanisms in statistical analyses to avoid biased inferences.

**Fig. 5:**
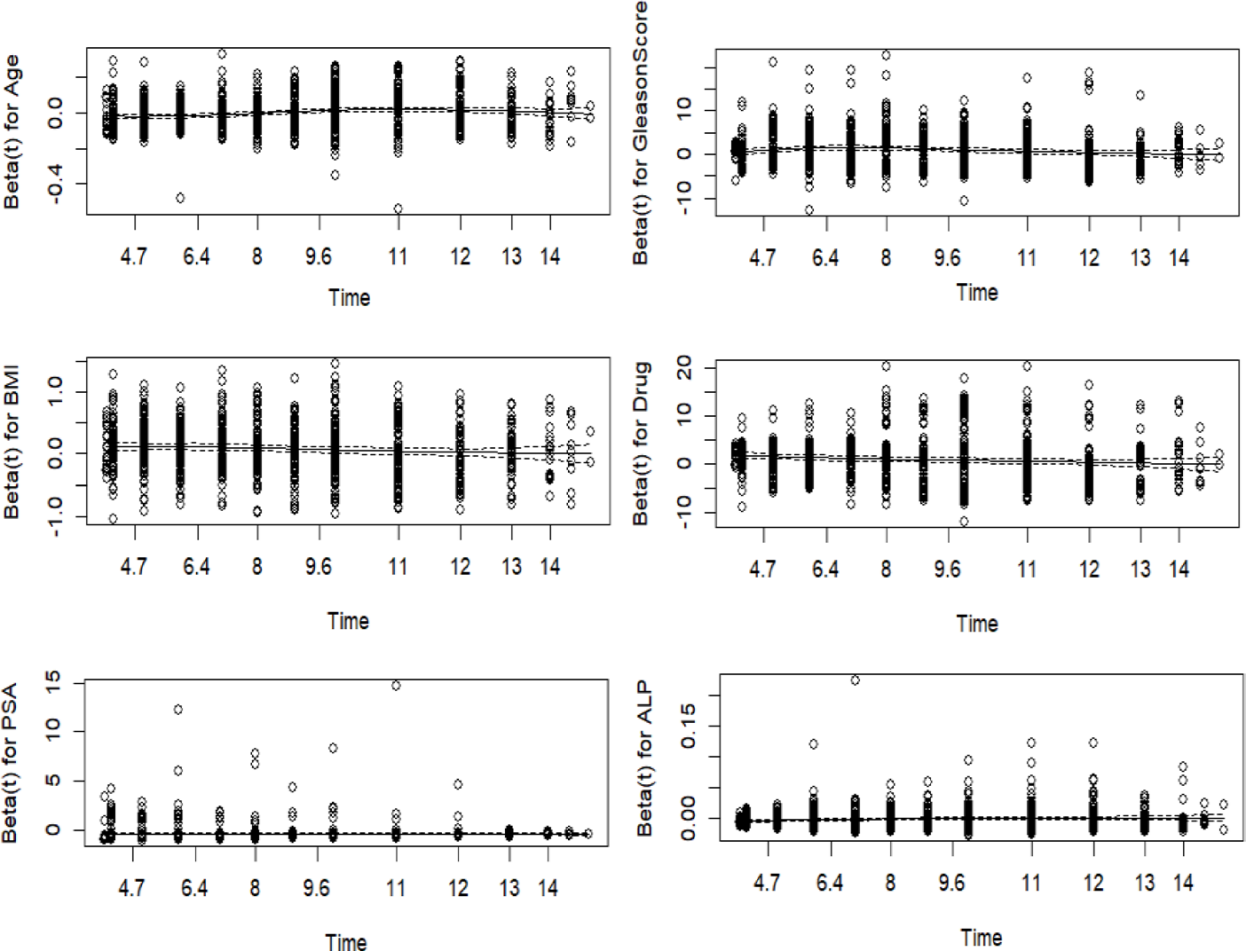
Scaled Schoenfeld residuals for individual variables, for the last imputed matrix.

Figure 5 presents Schoenfeld residual (S-R) plots assessing the proportional hazards (PH) assumption. Scaled residuals show no significant time-dependent deviations, confirming the adequacy of the PH model. The stability of HRs across imputed datasets, as demonstrated by Rubin’s rule combination, further supports the consistency of our findings.

In this figure, the fit lines for the scaled S-R are observed to be nearly horizontal, showing no significant deviations. This alignment suggests that the PH assumption holds for the model. In other words, the relationship between covariates and the hazard function remains stable over time, supporting the validity of the Cox PH model for the data being analyzed. The absence of substantial deviations from a horizontal line in the residual plots indicates that the model’s assumptions are appropriate, ensuring that the results from the event-time analysis are reliable. This confirms that the HR remains proportional over time, validating the use of the Cox PH model for PC data.

Figure 5 presents the results for the last imputed dataset, assessing the PH assumption. Scaled S-R^29^ are plotted against time for each of the five models. The fit lines do not show significant departures from the expected horizontal trend, indicating that the PH assumption is valid.

**Fig. 6:**
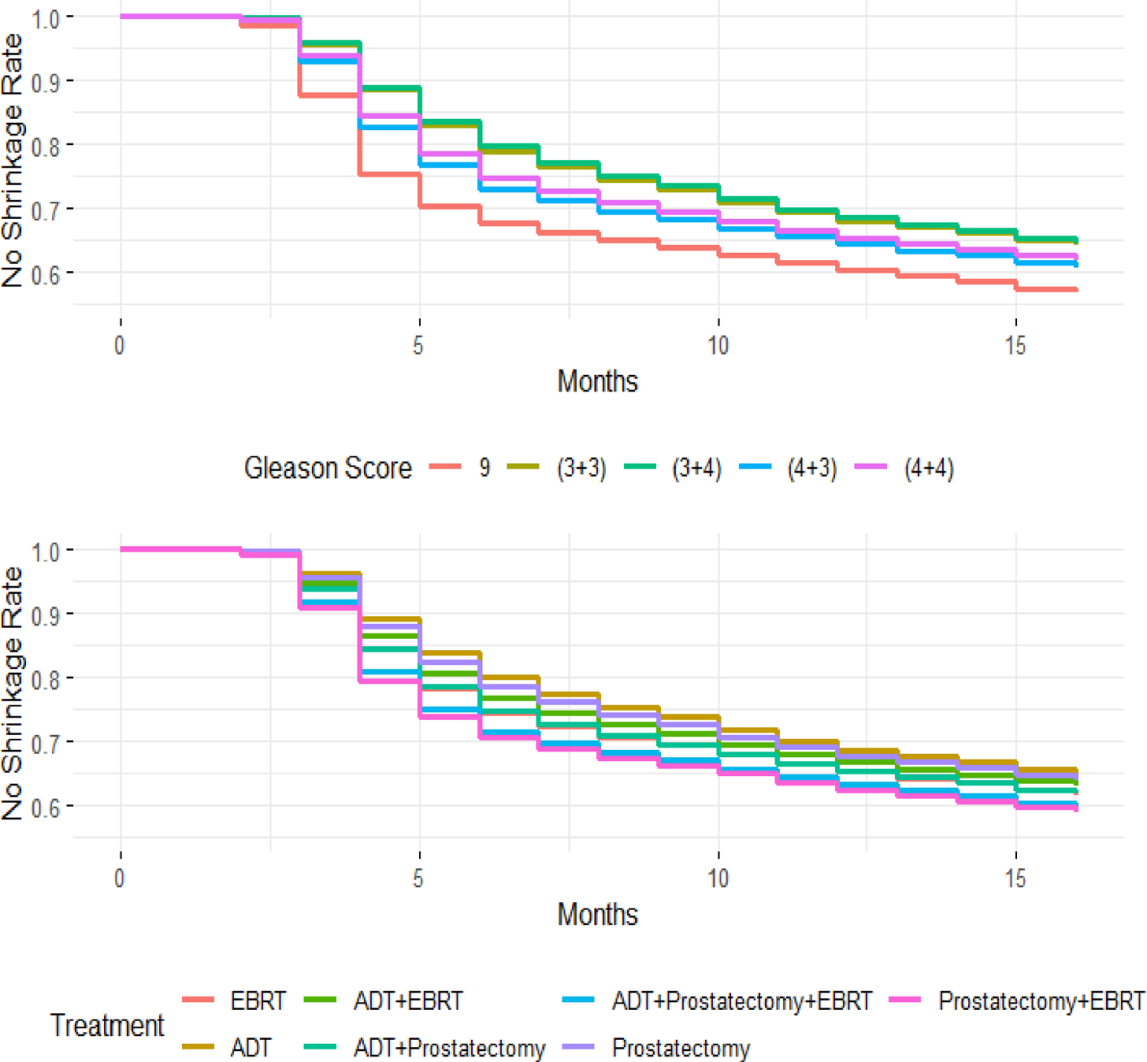
Cox adjusted survival curves for Gleason score and treatment.

Figure 6 displays survival curves (SC) for tumor non-reduction, adjusted for treatment category and Gleason Score using the Cox PH model. These curves illustrate the probability of tumor non-reduction over time, comparing different treatment groups and Gleason Score categories. Adjusting for covariates allows for more precise comparisons. Higher Gleason Scores are generally associated with a greater likelihood of tumor non-reduction, suggesting more aggressive tumors and a potentially poorer prognosis.

The comparison of survival curves (SC) across different treatment categories helps assess treatment effectiveness. Significant differences between these curves indicate that certain treatments are more effective in managing tumor progression. By adjusting for treatment type and Gleason Score, the analysis ensures accurate comparisons, highlighting the impact of tumor aggressiveness and treatment strategies on patient outcomes.

The Gleason Score is a crucial metric for evaluating PCaggressiveness, with higher scores indicating more aggressive tumors. Tumors with higher Gleason Scores typically exhibit a greater likelihood of non-reduction, which correlates with poorer prognostic outcomes. Understanding this metric allows clinicians to tailor treatment strategies based on disease severity.

Treatment approaches for PC vary depending on clinical scenarios and tumor characteristics. EBRT is commonly used to target localized PC, delivering high-energy radiation to destroy cancer cells. ADT works by reducing male hormone levels, which can slow or shrink hormone-sensitive tumors. Prostatectomy, a surgical procedure to remove the prostate gland, is often considered for patients with localized but high-risk disease.

In high-risk cases, combination therapies are frequently recommended to enhance treatment efficacy. For example, integrating EBRT with ADT or combining prostatectomy with radiation therapy can improve patient outcomes by addressing both local and systemic disease components. In severe or advanced cases, a multimodal approach incorporating EBRT, ADT, and prostatectomy may provide the most comprehensive treatment strategy. Each treatment plan is carefully tailored based on the Gleason Score, cancer stage, and individual patient needs to optimize therapeutic outcomes.

Table 2 presents the results of the Cox PH model applied after MI to address missing data. The model has evaluated the effect of key covariates on time to tumor shrinkage, reporting estimated coefficients, Standard Error (Std error), HR, and 95% CI. Age has not significantly impacted tumor shrinkage (HR = 0.998, *p* = 0.711), suggesting that within this cohort, age is not a key determinant of treatment response. BMI, however, has been significantly associated with tumor shrinkage (HR = 1.084, *p* < 0.001), indicating that higher BMI has increased the hazard of tumor shrinkage. Compared to patients with a Gleason Score of 9, lower Gleason Scores have been significantly associated with an increased hazard of tumor shrinkage (*p* < 0.001 for most categories).The strongest effect is observed for Gleason 3 + 3 (HR = 0.278, *p* < 0.001) and Gleason 3 + 4 (HR = 0.260, *p* < 0.001), indicating that patients with less aggressive tumors have a higher likelihood of tumor shrinkage compared to those with Gleason 9.Patients with Gleason 4 + 3 (HR = 0.499, *p* = 0.003) and Gleason 4 + 4 (HR = 0.419, *p* < 0.001) have also significantly increased hazard of shrinkage, though the effect is less pronounced than for lower Gleason Scores.Compared to EBRT (reference group), ADT aloneissignificantly associated with a reduced hazard of tumor shrinkage (HR = 0.592, *p* = 0.006), indicating that EBRT alone is less effective in inducing tumor shrinkage.ADT + EBRT (HR = 0.795, *p* = 0.234)and ADT + Prostatectomy (HR = 0.983, *p* = 0.950)are not shown statistically significant effects.Prostatectomy + EBRT i**s**the only treatment associated with an increased hazard of tumor shrinkage (HR = 1.578, *p* = 0.028), suggesting that combined surgical and radiotherapy intervention has improved treatment response.

**Table 2:** Cox regression model with MI fortumor shrinkage.

Covariates	Coefficient	Stderror	*p*-value	HR	HR 95% CI	
					Lower	Upper
Age	− 0.002	0.005	0.711	0.998	0.989	1.008
Gleason Score (ref = 9)						
(3 + 3)	− 1.281	0.287	0.000	0.278	0.158	0.488
(3 + 4)	− 1.348	0.280	0.000	0.260	0.150	0.450
(4 + 3)	− 0.696	0.231	0.003	0.499	0.317	0.784
(4 + 4)	− 0.871	0.224	0.000	0.419	0.270	0.650
BMI	0.080	0.019	0.000	1.084	1.045	1.124
Treatment (ref = EBRT)						
ADT	− 0.524	0.190	0.006	0.592	0.408	0.858
ADT + EBRT	− 0.229	0.192	0.234	0.795	0.545	1.160
ADT + prostatectomy	− 0.017	0.273	0.950	0.983	0.576	1.677
ADT + prostatectomy + EBRT	0.330	0.383	0.389	1.391	0.657	2.947
Prostatectomy	− 0.390	0.239	0.103	0.677	0.424	1.082
Prostatectomy + EBRT	0.456	0.207	0.028	1.578	1.052	2.369
PSA	− 0.361	0.034	0.000	0.697	0.653	0.745
AlkalinePhosphatase	− 0.001	0.000	0.042	0.999	0.998	1.000

PSA levels are strongly associated with tumor shrinkage, with a lower PSA corresponding to an increased likelihood of tumor shrinkage (HR = 0.697, *p* < 0.001). This suggests that higher PSA levels predicted poorer response to treatment.

Alkaline Phosphatase has shown a small but significant association with tumor shrinkage (HR = 0.999, *p* = 0.042), indicating that Alkaline Phosphatase’s higher levels have been linked to a slightly lower hazard of tumor shrinkage.

The Cox regression model demonstrates that Gleason Score, BMI, PSA levels, and treatment typearesignificant predictors of tumor shrinkage. Patients with higher Gleason Scores and elevated PSA levels have poorer treatment responses, while those receiving prostatectomy combined with radiotherapy have exhibited better outcomes. Additionally, the small but significant effect of Alkaline Phosphatasehas suggested a potential role in predicting tumor response, warranting further investigation.

### Sensitivity analysis

To address the challenge of missing data, sensitivity parameters are introduced. These parameters, which are functions of model variables, influence the extrapolation distribution and play a crucial role in understanding how assumptions about missingness affect the final analysis.

To study the influence of missingness on final inferences, sensitivity analysis is conducted using the DA technique^30^. Two default values (-2 and − 1) and two excess values (1 and 2) are selected for the DA coefficient, with 0 representing the MAR scenario. These values are chosen based on the logarithm of Alkaline Phosphatase and its variability. Table 3 presents the results of fitting a Cox model using imputed and perturbed data for each delta value.

**Table 3:** Hazard ratio (HR) and 95% confidence interval (CI) from the Cox model using the imputed and disturbed data according to each delta value.

Variable	Delta				
	− 2	− 1	0 (MAR)	1	2
Age	0.999(0.990;1.009)	1.000 (0.990; 1.009)	0.998 (0.989; 1.007)	0.997 (0.988; 1.006)	0.998 (0.989; 1.007)
Gleason Score (ref = 9)					
(3 + 3)	0.224 (0.127; 0.397)	0.231 (0.131; 0.406)	0.277 (0.158; 0.486)	0.285 (0.162; 0.501)	0.279 (0.159; 0.492)
(3 + 4)	0.209 (0.120; 0.366)	0.216 (0.124; 0.376)	0.260 (0.105; 0.405)	0.262 (0.151; 0.455)	0.256 (0.147; 0.445)
(4 + 3)	0.433 (0.274; 0.682)	0.441 (0.281; 0.693)	0.497 (0.317; 0.781)	0.506 (0.320; 0.799)	0.502 (0.317; 0.795)
(4 + 4)	0.365 (0.234; 0.570)	0.372 (0.239; 0.579)	0.417 (0.269; 0.646)	0.416 (0.266; 0.649)	0.409 (0.261; 0.641)
BMI	1.086 (1.048; 1.126)	1.084 (1.045; 1.124)	1.083 (1.045; 1.123)	1.091 (1.052; 1.130)	1.093 (1.054; 1.133)
Treatment (ref = EBRT)					
ADT	0.554 (0.382; 0.802)	0.556 (0.384; 0.805)	0.592 (0.409; 0.858)	0.609 (0.422; 0.878)	0.602 (0.417; 0.868)
ADT + EBRT	0.687 (0.470; 1.005)	0.705 (0.483; 1.030)	0.799 (0.549; 1.164)	0.814 (0.560; 1.182)	0.790 (0.543; 1.149)
ADT+ Prostatectomy	0.862 (0.507; 1.465)	0.884 (0.522; 1.499)	0.993 (0.583; 1.693)	1.021 (0.599; 1.742)	0.988 (0.576; 1.695)
ADT+ prostatectomy + EBRT	1.115 (0.535; 2.322)	1.181 (0.560; 2.492)	1.377 (0.650; 2.917)	1.386 (0.661; 2.906)	1.314 (0.625; 2.765)
Prostatectomy	0.651 (0.414; 1.022)	0.656 (0.416; 1.034)	0.682 (0.427; 1.089)	0.693 (0.437; 1.101)	0.688 (0.435; 1.090)
Prostatectomy + EBRT	1.312 (0.871; 1.974)	1.356 (0.903; 2.037)	1.592 (1.063; 2.384)	1.632 (1.086; 2.45)	1.569 (1.042; 2.362)
PSA	0.700 (0.655; 0.748)	0.700 (0.654; 0.748)	0.697 (0.653; 0.745)	0.698 (0.654; 0.745)	0.699 (0.654; 0.746)
AlkalinePhosphatase	0.995 (0.992; 0.997)	0.995 (0.992; 0.997)	0.998 (0.997; 1.000)	1.000 (0.999; 1.001)	1.000 (1.000; 1.001)

Table 3 presents the HR estimates and 95% CI from the Cox PH model MI and DA imputed datasets. The sensitivity analysis is performed across different delta values (-2, -1, 0, 1, 2), where delta represents the degree of deviation from the MAR assumption.

Across all delta values, age is not significantly associated with tumor shrinkage, with HR values close to 1 and CI consistently spanning 1. This suggests that within the study population, age has not been a major determinant of treatment response regardless of the missing data assumptions.

Compared to patients with a Gleason Score of 9, those with lower Gleason Scores have exhibited a higher likelihood of tumor shrinkage, as indicated by HR below 1 across all delta values. The effect remains robust across different delta adjustments, with slight variations in HR estimates. Gleason 3 + 3: HR ranges from 0.224 (-2) to 0.279 (2), showing a significant increase in tumor shrinkage likelihood for lower Gleason Scores. For the Gleason Score 3 + 4 h ranges from 0.209 (-2) to 0.256 (2), confirming a consistent trend.GleasonScores 4 + 3 and 4 + 4also show an increased hazard of tumor shrinkage, although the effect is less pronounced than in lower-grade tumors.

BMI has consistently exhibited a significant positive association with tumor shrinkage across all delta values. The HRs have remained above 1, ranging from 1.083 (MAR) to 1.093 (2), indicating that higher BMI is linked to an increased probability of tumor shrinkage.

Compared to EBRT (reference group), ADT treatmentaloneisconsistently associated with a reduced hazard of tumor shrinkage (HR = 0.592 at MAR), with HRs increasing slightly as the delta increases.ADT + EBRTdoes not show statistically significant effects, with HRs ranging from 0.687 (-2) to 0.790 (2).ADT + Prostatectomy remained non-significant across delta values, suggesting that this combination does not provide a clear survival benefit.Prostatectomy + EBRT has been the only treatment associated with a significantly increased hazard of tumor shrinkage at MAR (HR = 1.592, 95% CI: 1.063–2.384), and the association has remained robust across delta values.PSA levels have consistently shown a strong inverse association with tumor shrinkage (HR = 0.697 at MAR), suggesting that higher PSA levels have been predictive of a lower probability of tumor shrinkage.Alkaline Phosphatasehas exhibited a weaker, yet significant, association with tumor shrinkage. The effect has varied across delta values, with HRs shifting from 0.995 (-2) to 1.000 (2), indicating a potential impact on treatment response.

The results from Table 3 have demonstrated that Gleason Score, BMI, PSA levels, and treatment type have been significant predictors of tumor shrinkage, with associations remaining stable across different missing data assumptions. Prostatectomy combined with radiotherapy has been the only treatment consistently associated with improved outcomes. These findings underscore the importance of considering missing data mechanisms in survival analysis to ensure the robustness of conclusions drawn from imputed datasets.

Data analysis is performed using RStudio v. 2023.12.0, with the following R packages utilized for data manipulation, imputation, and survival analysis: dplyr^31^, DataExplorer^32^, mice^33^, survminer^34^, and survival^35^.

## Discussion

This study investigates the impact of missing data on prognostic modeling in PCby applying ignorability principles and DA under the MI framework. The key findings indicate that Alkaline Phosphatase is a significant biomarker for tumor shrinkage in PC patients, with its prognostic value being highly sensitive to missing data assumptions. PSA consistently shows a negative association with tumor shrinkage hazard, reinforcing its role as a predictive biomarker. The Gleason Score remains a strong predictor, with lower scores (e.g., 3 + 3 and 3 + 4) associated with a reduced hazard of tumor shrinkage. Additionally, higher BMI is positively linked to tumor shrinkage, while ADT alone is associated with a lower hazard of tumor shrinkage compared to combination therapies. These results provide important insights into the validity of missing data assumptions and their influence on key prognostic factors in PC.

Our findings align with previous research on the prognostic value of Alkaline Phosphataseand PSA in PC. Studies have demonstrated that elevated. Alkaline Phosphatase levels are indicative of bone metastasis and aggressive disease progression. The observed sensitivity ofAlkaline Phosphatase’s HR to missingness assumptions underscores the importance of robust missing data handling techniques, particularly when estimating treatment effects in longitudinal cancer studies^27^. Similarly, PSA has long been recognized as a critical biomarker in PC prognosis, and our findings reinforce its negative association with tumor shrinkage hazard. The observed association between lower Gleason Scores and reduced hazard of tumor shrinkage is consistent with existing evidence indicating that higher Gleason Scores correlate with more aggressive disease progression and poorer treatment outcomes^25^.

Moreover, our findings highlight BMI’s potential role in PC prognosis. While previous studies have reported mixed results regarding the association between BMI and PC progression, our study suggests that higher BMI may be linked to an increased likelihood of tumor shrinkage. This emphasizes the need for further investigation into the metabolic and treatment-related factors influencing this association. Regarding treatment effects, our study shows that ADT alone is associated with a lower hazard of tumor shrinkage, as evidenced by HR values below 1 across all delta values. However, combination treatments such as prostatectomy and EBRT exhibit varying degrees of association, with some combinations indicating a higher HR, particularly under the MAR assumption.

Despite the strengths of our data analysis approach to missing data analysis, several limitations should be acknowledged. A high percentage of missing data for key biomarkers such asAlkaline Phosphatase and PSA poses challenges, as extreme missingness (> 50%) can introduce bias. Although our MI framework accounts for missingness under both MAR and NMAR assumptions, future studies should incorporate simulation-based sensitivity analyses to further assess the robustness of imputations^36^. Additionally, the study is based on patients from Mayo Hospital, Lahore, which may limit generalizability. Future research should validate findings using multi-center datasets. Lastly, this study focuses on a single outcome tumor shrinkage. Incorporating additional clinical endpoints, such as progression-free survival or overall survival, could provide a more comprehensive understanding of treatment effectiveness^37^.

This study demonstrates the importance of rigorous missing data handling in the analysis of PC biomarkers, particularly for Alkaline Phosphatase. Given the sensitivity ofAlkaline Phosphatase’s prognostic value to missing data, further research is warranted to develop robust imputation strategies and validate these findings in larger, multi-center datasets. By refining statistical methodologies for handling missing data in oncology research, this study contributes to more reliable prognostic modeling and enhanced clinical decision-making in PC management.

## Electronic supplementary material

Below is the link to the electronic supplementary material.

Supplementary Material 1

## Data Availability

The datasets used and/or analysed during the current study available from the corresponding author on reasonable request.

## References

[1] Pedersen, A. B. et al. Missing data and multiple imputation in clinical epidemiological research. *Clin. Epidemiol.* **9**, 157–166 (2017). 28352203 10.2147/CLEP.S129785 PMC5358992

[2] Powney, M. et al. A review of the handling of missing longitudinal outcome data in clinical trials. *Trials*. **15**, 237 (2014). 10.1186/1745-6215-15-237 PMC4087243 24947664

[3] Little, R. J. A class of pattern-mixture models for normal incomplete data. *Biometrika* **81**, 471–483 (1994).

[4] Ibrahim, J. G., Chu, H. & Chen, M. H. Missing data in clinical studies: issues and methods. *J. Clin. Oncol.* **30**, 3297–3303 (2012). 22649133 10.1200/JCO.2011.38.7589 PMC3948388

[5] Schafer, J. L. Multiple imputation: A primer. *Stat. Methods Med. Res.* **8**, 3–15 (1999). 10347857 10.1177/096228029900800102

[6] Piedvache, A. et al. Strategies for assessing the impact of loss to follow-up on estimates of neurodevelopmental impairment. *BMC Med. Res. Methodol.* **21**, 118 (2021). 34092226 10.1186/s12874-021-01264-3 PMC8182922

[7] Graham, J. W. *Missing Data: Analysis and Design* (Springer, 2012).

[8] Cro, S. et al. Sensitivity analysis for clinical trials with missing continuous outcome data using controlled multiple imputation: A practical guide. *Stat. Med.* **39**, 2815–2842 (2020). 32419182 10.1002/sim.8569

[9] Howe, C. J. et al. Selection bias due to loss to follow-up in cohort studies. *Epidemiology* **27**, 91–97 (2016). 26484424 10.1097/EDE.0000000000000409 PMC5008911

[10] Westreich, D. Berkson’s bias, selection bias, and missing data. *Epidemiology* **23**, 159–164 (2012). 22081062 10.1097/EDE.0b013e31823b6296 PMC3237868

[11] Javanbakht, M. et al. Comparing single and multiple imputation strategies for harmonizing substance use data across HIV-related cohort studies. *BMC Med. Res. Methodol.* **22**, 90 (2022). 35369872 10.1186/s12874-022-01554-4 PMC8978400

[12] Van Buuren, S., Boshuizen, H. C. & &Knook, D. L. Multiple imputation of missing blood pressure covariates in survival analysis. *Stat. Med.* **18**, 681–694 (1999). 10204197 10.1002/(sici)1097-0258(19990330)18:6<681::aid-sim71>3.0.co;2-r

[13] Clark, T. G. & Altman, D. G. Developing a prognostic model in the presence of missing data: an ovarian cancer case study. *J. Clin. Epidemiol.* **56**, 28–37 (2003). 12589867 10.1016/s0895-4356(02)00539-5

[14] Barzi, F. & Woodward, M. Imputations of missing values in practice: results from imputations of serum cholesterol in 28 cohort studies. *Am. J. Epidemiol.* **160**, 34–45 (2004). 15229115 10.1093/aje/kwh175

[15] Burne, R. M. & Abrahamowicz, M. Martingale residual-based method to control for confounders measured only in a validation sample in time‐to‐event analysis. *Stat. Med.* **35**, 4588–4606 (2016). 27306611 10.1002/sim.7012

[16] Little, R. J. & Rubin, D. B. *Statistical Analysis with Missing Data* Vol. 793 (Wiley, 2019).

[17] Carpenter, J. R. et al. *Multiple Imputation and its Application* (Wiley, 2023).

[18] Rubin, D. B. *Multiple Imputation for Nonresponse in Surveys* (Wiley, 1987).

[19] Leacy, F. P. et al. Analyses of sensitivity to the missing-at-random assumption using multiple imputation with delta adjustment. *Am. J. Epidemiol.* **185**, 304–315 (2017). 28073767 10.1093/aje/kww107 PMC5860630

[20] Leurent, B. et al. Sensitivity analysis for not-at-random missing data in trial-based cost-effectiveness analysis: A tutorial. *Pharmacoeconomics* **36**, 889–901 (2018). 29679317 10.1007/s40273-018-0650-5 PMC6021473

[21] Heckman, J. J. Sample selection bias as a specification error. *Econometrica*. **47**, 153–161 (1979).

[22] Zhao, Y. et al. A multiple imputation method for sensitivity analyses of time-to-event data. *J. Biopharm. Stat.* **24**, 229–253 (2014). 24605967 10.1080/10543406.2013.860769 PMC4009741

[23] Cox, D. R. Regression models and life-tables. *J. R Stat. Soc.* **B34**, 187–202 (1972).

[24] White, H. K. et al. Impact of the deepwater horizon oil spill on a deep-water coral community in the Gulf of Mexico. *Proc. Natl. Acad. Sci.* **109**(50), 20303–20308 (2012). 10.1073/pnas.1118029109 PMC3528508 22454495

[25] Liaqat, M. et al. Relationship between prostate-specific antigen, alkaline phosphatase levels, and time-to-tumor shrinkage. *BMC Urol.* **24**, 137 (2024). 38956570 10.1186/s12894-024-01522-8 PMC11221162

[26] Liaqat, M., Kamal, S. & Fischer, F. Illustration of association between change in prostate-specific antigen values and time to tumor status. *BMC Urol.* **23**, 202 (2023). 38057759 10.1186/s12894-023-01374-8 PMC10702046

[27] Van Buuren, S. & Groothuis-Oudshoorn, K. Mice: multivariate imputation by chained equations in R. *J. Stat. Softw.* **45**, 1–67 (2011).

[28] White, I. R. & Royston, P. Imputing missing covariate values for the Cox model. *Stat. Med.* **28**, 1982–1998 (2009). 19452569 10.1002/sim.3618 PMC2998703

[29] Schoenfeld, D. Partial residuals for the proportional hazards regression model. *Biometrika* **69**, 239–241 (1982).

[30] Rezvan, P. H., Lee, K. J. & Simpson, J. A. Sensitivity analysis within multiple imputation framework using delta-adjustment. *Longit Life Course Stud.* **9**, 259–278 (2018).

[31] Wickham, H. & Francois, R. *dplyr: A grammar of data manipulation*. R package version 1.0. https://cran.r-project.org/web/packages/dplyr/ (2023).

[32] Zhou, S. & DataExplorer Exploratory data analysis. R package version 1.0. https://cran.r-project.org/web/packages/DataExplorer/ (2023).

[33] van Buuren, S. et al. mice: Multivariate Imputation by Chained Equations (version 3.16.0) [Computer software] (2023).

[34] Kassambara, A., Daniels, M. J. & Hogan, J. W. *survminer: Drawing survival curves*. R package version 0.4.9. https://cran.r-project.org/web/packages/survminer (2023).

[35] Therneau, T., & Atkinson, E. The concordance statistic. *A package for survival analysis in R, vignettes*. R package version, 3-7 (2023).

[36] Daniels, M. J. & Hogan, J. W. *Missing Data in Longitudinal studies: Strategies for Bayesian Modeling and Sensitivity Analysis*. (CRC Press, 2008).

[37] Moreno-Betancur, M. & &Chavance, M. Sensitivity analysis of incomplete longitudinal data. *Stat. Methods Med. Res.* **25**, 1471–1489 (2016). 23698867 10.1177/0962280213490014

