# Supplementary material for: Sensitivity analysis for time-to-event data accounting for intra-individual variability in time-varying covariates with missing data
Source: Sci Rep. 2025 Jul 24;15:27012. doi: 10.1038/s41598-025-09599-3 (PMC12289879; doi:10.1038/s41598-025-09599-3)
Supplement: Supplementary file 1 — Supplementary Material 1 [file 41598_2025_9599_MOESM1_ESM.docx]

**Delta-Adjusted Multiple Imputation (DA-MI) Algorithm**

**Step 1: Define the Data Structure**

- Y=log(ALP): the outcome variable with missing values (log-transformed Alkaline Phosphatase).
- X={Age, Gleason Score, BMI, Drug, PSA}: fully observed covariates, where PSA is time-dependent and the rest are baseline covariates.
- X_obs_​: observed values of log(ALP).
- X_mis_​: missing values of log(ALP).

**Step 2: Specify the Imputation Model under MAR**

P(log(ALP)∣Age,Gleason Score,BMI,Drug,PSA;θ)

where θ represents parameters estimated under the MAR assumption.

**Step 3: Introduce the Sensitivity Parameter δ**

We define a scalar sensitivity parameter δ, which shifts the imputed values:

$${log(ALP)}_{mis}^{\delta}={log(ALP)}_{mis}^{\mathrm{MAR}}+\delta$$

**Step 4: Perform Multiple Imputations with Delta Adjustment**

For each of the m=1,…,M imputations:

1. Estimate model parameters θ^(m).
2. Generate imputed values under MAR:
3. ${log(ALP)}_{mis}^{\delta}\sim P(\cdot\mid X_{\mathrm{obs}},\theta^(m)).$
4. ${log(ALP)}_{mis}^{m,\delta}={log(ALP)}_{mis}^{m}+\delta$.

**Step 5: Fit the Analysis Model**

For each of the M imputed datasets, fit a Cox proportional hazards model that includes:

- Baseline covariates: Age, Gleason Score, BMI, Drug
- Time-dependent covariates: PSA, log(ALP)

**Step 6: Pool Estimates Using Rubin’s Rules**

For each parameter:

1. Compute the pooled estimate ​ across imputations.
2. Compute within-imputation variance and between-imputation variance.
3. Calculate total variance.
4. Construct confidence intervals and conduct inference using pooled estimates.

**Step 7: Repeat for a Range of δ**

Repeat Steps 4 to 6 for a range of sensitivity parameters δ=−2,−1,0,1,2 to evaluate how inferences vary with assumptions about the missing data mechanism.

**Step 8: Interpretation**

Analyze parameter estimates and confidence intervals change with different δ values.

**R codes of present study :**

**# Load packages**

**suppressPackageStartupMessages({**

**library(mice)**

**library(survival)**

**library(dplyr)**

**library(purrr)**

**})**

**# Create working copy and transform ALP → log scale only for imputation**

**data_work<- data_raw %>%**

**mutate(logALP = ifelse(!is.na(ALP), log(ALP), NA)) %>%**

**select(-ALP)**

**# Function to perform mice under MAR**

**get_mice_imp<- function(data, seed = 123) {**

**mice(**

**data,**

**m = 5,**

**maxit = 5000,**

**seed = seed,**

**method = "norm.predict" # Imputation method for continuous variables**

**)**

**}**

**# Function to apply delta-adjustment and back-transform logALP to ALP**

**adjust_delta<- function(imp_obj, delta) {**

**imputed_datasets<- complete(imp_obj, "all")**

**map(imputed_datasets, function(df) {**

**imp_index<- which(is.na(data_work$logALP))**

**df$logALP[imp_index] <- df$logALP[imp_index] + delta**

**df$ALP<- exp(df$logALP)**

**df # return modified dataframe**

**})**

**}**

**# Function to fit Cox model on a dataset**

**fit_cox<- function(df) {**

**coxph(Surv(time, status) ~ Age + BMI + Gleason + Drug + PSA + ALP, data = df)**

**}**

**# Full delta sensitivity pipeline**

**run_da_mi<- function(deltas = c(-2, -1, 0, 1, 2)) {**

**imp_base<- get_mice_imp(data_work)**

**results<- list()**

**for (d in deltas) {**

**imp_dsets<- adjust_delta(imp_base, d)**

**model_fits<- map(imp_dsets, fit_cox)**

**pooled<- pool(model_fits)**

**results[[paste0("delta=", d)]] <- summary(pooled)**

**}**

**return(results)**

**}**

**# Run and inspect results**

**delta_results<- run_da_mi()**
